# Radon Sources and Associated Risk in Terms of Exposure and Dose

**DOI:** 10.3389/fpubh.2014.00207

**Published:** 2015-01-05

**Authors:** Efstratios G. Vogiannis, Dimitrios Nikolopoulos

**Affiliations:** ^1^Evangeliki Model School of Smyrna, Nea Smyrni, Greece; ^2^Department of Computer and Electronic Engineering, Technological Education Institute of Pireaus, Aigaleo, Greece

**Keywords:** radon, progeny, human health, exposure, dose, lung cancer, radon risk and indoor air

## Abstract

Radon concerns the international scientific community from the early twentieth century, initially as radium emanation and nearly the second half of the century as a significant hazard to human health. The initial brilliant period of its use as medicine was followed by a period of intense concern for its health effects. Miners in Europe and later in the U.S were the primary target groups surveyed. Nowadays, there is a concrete evidence that radon and its progeny can cause lung cancer (1). Human activities may create or modify pathways increasing indoor radon concentration compared to outdoor background. These pathways can be controlled by preventive and corrective actions (2). Indoor radon and its short-lived progeny either attached on aerosol particles or free, compose an air mixture that carries a significant energy amount [Potential Alpha-Energy Concentration (PAEC)]. Prior research at that topic focused on the exposure on PAEC and the dose delivered by the human body or tissues. Special mention was made to the case of water workers due to inadequate data. Furthermore, radon risk assessment and relevant legislation for the dose delivered by man from radon and its progeny has been also reviewed.

## Introduction

Naturally occurring radioactive materials (NORMs) are present in human radiation environment. In recent years, considerable attention has been paid to radon, which is a natural, colorless, odorless, and tasteless radioactive noble gas. Three are the main naturally occurring isotopes of radon, ^222^Rn, ^220^Rn, and ^219^Rn. ^222^Rn is the direct descendent of ^226^Ra. Both of them are members of the uranium (4n + 2) series. ^220^Rn is also known as thoron (Tn) because it is a member of the thorium (4n) series. All radon isotopes are NORMs. However, most of the radioactivity in the atmosphere at sea level is attributable to ^220^Rn and, mostly, ^222^Rn. ^222^Rn importance is due to its high abundance of weight (99.27%) compared to the total natural mixture of all radon isotopes. For that reason, the term “radon” identifies mainly the ^222^Rn and this is the convention followed thereafter. Radon (^222^Rn) is released primarily from soil and approximately 10% of it is released into the atmosphere (3, 4).

^218^Po, ^214^Pb, and ^214^Bi are radon’s most significant progeny in terms of radiation dose. Radon and progeny interact indoors with aerosol particles via complex physical phenomena. These produce an inhalable radioactive mixture indoors. Radon progeny appears in two forms; attached on aerosol particles and unattached. Most of the activity of radon progeny is associated with particles of small diameter between 0.006 and 0.2 mm, with mean diameter of about 0.025 mm. A small fraction of radon progeny, typically 0.1 or less, remains unattached and in dynamic equilibrium with attached particles. Generally, dustier atmospheres are associated with smaller values of unattached fraction and higher concentrations of radon due to additional radiation emission from dust. Attached radon progeny in the air settle to earth by gravity and other processes (3). Some attached particles plate-out on surfaces (3).

Concentration of radon and progeny vary considerably due to concentration variations of ^226^Ra and ^232^Th present in soil. Atmospheric concentrations are greater over areas in which soil is rich in ^226^Ra and ^232^Th. The corresponding concentrations are lower over oceans and large water tanks because of lower concentrations of ^226^Ra. Various factors affect ^222^Rn concentrations in air: (1) ground cover (e.g., paving, buildings, and vegetation); (2) altitude or height above ground; (3) soil porosity and grain size; (4) temperature; (5) atmospheric pressure; (6) soil moisture, rainfall, and snow cover; (7) atmospheric conditions; and (8) season. Seasonal and diurnal variations in atmospheric radon concentrations vary with geographic location. Globally, ground level concentrations are maximal during autumn and early winter, and minimal during spring (5). Diurnally, radon concentrations are usually greatest during early morning because atmospheric temperature inversions are more likely to occur. After sunrise, heating causes inversion to burn off, allowing radon to rise by occupying larger volumes. Radon concentrations are usually low in the afternoon. Toward sunset, concentrations increase as the earth cools and atmospheric stability increases. Diurnal variation in concentration is typically within a range of factor of three to five, but variations of >10 have been reported at some locations. (6). Ground level ^222^Rn measurements indicate average concentrations of about 5–10 Bq/m^3^. There are also certain locations near earth’s surface with very high atmospheric concentrations of ^222^Rn and progeny; even several orders of magnitude higher than the averages. Generally, ^222^Rn concentrations range between 0.7 and 35 Bq/m^3^, with a mean of about 7 Bq/m^3^. Antarctica presents quite lower ^222^Rn in air due to geophysical reasons (7). Islands such as Hawaii and Philippines present also lower atmospheric radon levels. In caves, mines, and other enclosed spaces concentrations are considerably higher because radon builds up. In the Carlsbad Caverns in New Mexico, concentrations as low as 2 Bq/m^3^ have been measured while levels as high as a few Megabecquerel per cubic meters have been reported in enclosed mines (3). Concentrations of ^220^Rn progeny are typically 3- to 10-fold lower than those of radon (6).

## Historical Aspects

In the early 1900s, the public showed interest in radium, which was considered at that time as a self-contained source of energy for cure. Thereafter and for about three decades, several radium-based medicines and nostrums made their appearance in pharmacies throughout the world. Radium-rich waters from spas and mineral springs were sold as tonics and for their hypothetical curative properties. Radium salts were listed in the *New and Non-official Remedies* (NNR) of the Council of Medicine and Pharmacy of the American Medical Association, and in a number of pharmacopeias in countries other than the United States (8). Chemist Frederick Soddy (1877–1956) proposed in 1905 air bubbling of radium solutions as a method to obtain radon gas, which was suggested as a treatment for tuberculosis. At that time, radon was proposed for several medical uses, as for example, insufflation into various body cavities. At spas, the practices were not limited to bathing and water consumption, but were extended to inhalation of radon collected in a specially enclosed room known as an emanatorium or inhalatorium (6). In 1951, the “Free Enterprise Mine” in Montana-U.S. begun operations as the radon exposure was considered beneficial by its founders for the treatment of arthritis, asthma, sinusitis, and similar ailments.

The practice of radon inhalation continues nowadays. Several mines with high radon levels radon are still accessible to the public. Several are found in Montana–U.S., in Czechoslovakia, Japan, Poland, and the Russian Commonwealth. Several spa centers are also under operation. A noteworthy example is the spa of Badgastein in Austria, in which underground chambers are fitted with bunk beds and attendants to care for the clientele (6).

### Indoor radon

After its generation in soil-rock grains, radon emanates partially. Emanated radon travels in existing pore’s space and migrates to short or long distances. Thereafter, it may enter in building structures via house pipes (e.g., for water and natural gas), or from building materials. The relative importance of these pathways depends on the circumstances; the soil input is the most significant (9).

#### Radon in buildings and working places

**Figure 1:**
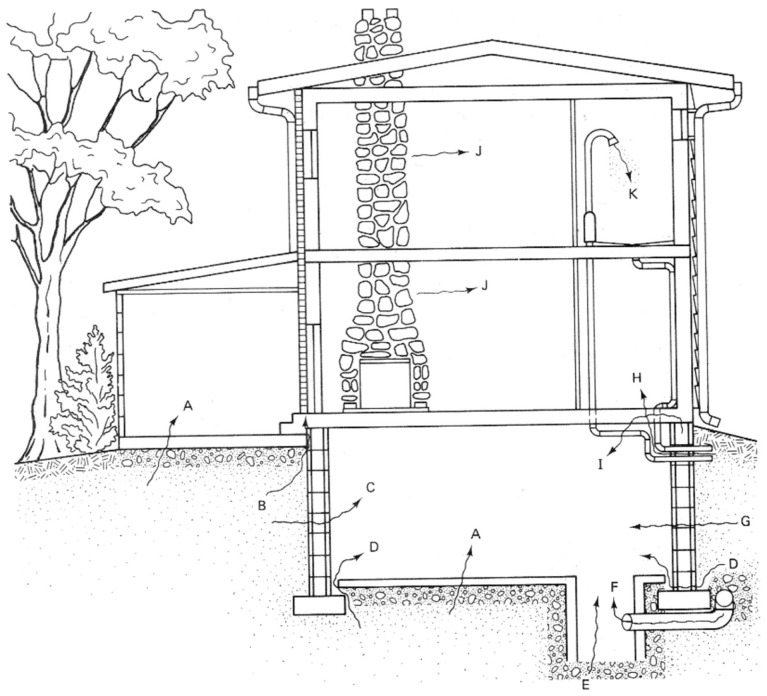
**Radon’s major entry points into a home**. A, cracks in concrete slabs; B, spaces behind brick veneer walls that rest on hollow – block foundation; C, pores and cracks in concrete blocks; D, floor–wall joints; E, exposed soil, as in a sump; F, weeping (drain) tile, if drained to popen sump; G, mortar joints; J, building materials, such as some rock; K, water (from some wells). Reproduced from Ref. (12).

Radon gas possible entry points into dwellings are illustrated in Figure 1. Understanding the radon mass balance for a building requires specific consideration of various sources. A median (or GM) entry rate for U.S single-family houses appears to be in the vicinity of 20 Bq m^−3^ h^−1^. Based on emanation rate measurements from U.S concretes, expected emissions should be approximately 2–3 Bq m^−3^ h^−1^ far below from the rate had been observed (10). On the other hand, the potential contribution from unattenuated soil flux with a median of 25 Bq m^−3^ h^−1^ corresponds closely with usual indoor observations (11). However, houses have understructures, which prevent radon’s entry, at least through diffusion, which is the main entrance mechanism. Building construction, nevertheless, is by far the main reason of radon’s entrance. As illustrated in Figure 1, the ground is the main source of radon’s entrance in buildings.

### Radon as a health hazard

#### Historical aspects

The adverse health effects of radon have been noticed since the fifteenth century. At that time, a German physician named Georgius Agricola (1494–1555), noted high fatality of miners due to lung diseases (8). Paracelsus (1493–1541) for more than 10 years surveyed the lung diseases occurred in many underground miners in the Erz Mountains of Eastern Europe. His research findings showed that the main reason for the deaths was the present of dust and gases in the mines (8). Later, the “Erz Mountain lung disease” was identified as lung cancer. Harting and Hess found in 1879 that approximately 75% of uranium miners of Germany and Czechoslovakia died unexpectedly (13). Later, Margaret Uhlig suggested that another possible cause of lung cancer is radium emanation (14). Between 1924 and 1932, it was hypothesized that radon exposure caused high rate of lung cancers among miners of Joachimstal in Chechoslovakia and Schneeberg in Germany (8). Pirchan and Sikl, concluded in 1932 that radium emanation causes lung tumors among the miners at Jachymov (8).Over half the deaths were from lung cancer and most occurred among miners before they reached the age of 50 years (8).

#### Studies of underground miners

Bale in 1951 (15) reported that lung cancer could occur due to inhaled alpha particles of radon progeny. However, the link between lung cancer incidence and high concentrations of radon progeny was scientifically accepted during 1990s after several investigations based on large epidemiological miner studies carried out in the decades 1950 and 1960. According to the Report VI of Committee on the Biological Effects of Ionizing Radiation (BEIR) (16), from a sample of 60,000 miners of the period 1941–1990 from 8 countries, over 2,600 developed lung cancer whereas only 750 were expected. Important investigations published by Lubin et al. (17–19), reported that 11 among 20 epidemiological studies of underground miners showed scientific evidence on linking radon exposure and lung cancer risk. The significant U.S Iowa study showed, in addition, high lung cancer mortality among women that were exposed to high residential radon levels (20). Today (2014), radon is considered as the second cause of lung cancer in the general population, after smoking (2, 21). According to World Health Organization (21), epidemiological studies have provided convincing evidence of an association between indoor radon exposure and lung cancer, even at the relatively low radon levels commonly found in residential buildings.

Radiation dose due to radon and progeny depends on concentration, particle size distribution, respiratory deposition, and lung clearance. Other important parameters affecting radiation dose are the morphometry of lungs and the breathing characteristics. The measurement of concentration of radon is, however, adequate only for estimating upper limits of exposure. Reliable measurements of radiation dose delivered from radon and its progeny must take into considerable account that the possible differentiations of the concentrations of radon progeny caused by various air conditions indoors. Although the typical equilibrium ratios between radon and progeny are the range between 0.4 and 0.5, upper and high extremes may be found (21). Nevertheless, the measurement of radon is preferred because it is simple and cost effective. Globally, radon can be considered as the radioactive pollutant associated with the majority of measurements. In the U.S alone, approximately one million indoor radon measurements are conducted annually (16, 22–26).

## Exposure to Radon and Its Decay Products

Radon and progeny accumulate indoors. Due to their radioactive properties, indoor air becomes a radiative mixture with significant amount of alpha-particle energy. Since ambient alpha-energy is a significant parameter in assessing energy deposited to lungs, different physical quantities have been introduced so-far [see e.g., Ref. (9)] in an effort to estimate related parameters. Some of these quantities are defined below.

The Equilibrium Equivalent Decay-product Concentration (EEDC) is defined as:

$${\mathrm{EEDC}}_{\mathrm{222}}=0.106C_{1}+0.\mathrm{513}C_{2}+0.\mathrm{381}C_{3}\;\mathrm{in}\;\mathrm{Bq}.m^{-3}$$

where C_1_, C_2_, and C_3_ are the concentrations of ^218^Po, ^214^Pb, and ^214^Bi. It calculates the concentration of an ideal mixture in which radon would be in radioactive equilibrium with its progeny.

The potential alpha-energy concentration (PAEC) describes the energy concentration carried by ambient radon and progeny and is calculated by the formula:

$$c_{p}^{x}=0.{\mathrm{58 C}}_{1}^{x}+2.{\mathrm{85 C}}_{2}^{x}+2.{\mathrm{1 C}}_{3}^{x}\;\;\mathrm{in}\;\mathrm{nJ}.m^{-3}$$

Superscript *x* denotes the form that progeny could be found i.e., *x* = *a* for progeny in attached form and *x* = *u* for progeny in unattached form, respectively (27). PAEC differentiates between attached and unattached progeny.

Unattached fraction $\mathrm{fp}\;=\;{c_{p}}^{u}∕\left({c_{p}}^{a}\;+\;{c_{p}}^{u}\right)$ identifies the fraction of energy delivered by tissues from unattached radon progeny. The unattached fraction (*f*_p_) comprises ultra-fine particles or clusters in the size range 0.5–5 nm (28, 29).

The total amount of alpha-energy carried in air is measured in Working Level (WL). 1 WL equals to 2.0810^−5^ J m^−3^ total energy amount radiated from all types of progeny.

The mean state of radioactive equilibrium between radon and its progeny is described by the equilibrium factor (*F*). *F*-factor is the ratio between the total PAEC per 55.9210^−10^ *C*_0_, where *C*_0_ is the radon concentration in ambient air. *F*-factor indicates the energy transferred from radon progeny in air related to the maximum potential energy that could be transferred. *F*-factor is a changeable factor depending on the internal space characteristics. It describes well the dynamic of the system. For example, if radon enters rapidly in a space, *F* decreases significantly until the time that equilibrium between radon and progeny is achieved, where *F* returns to the usual value of 0.4 adopted indoors.

Exposure is calculated as:

$$E=c_{p}*t$$

where *t* is the time of exposure over a tissue and *c*_p_ equals ${c_{p}}^{a}\;+\;{c_{p}}^{u}$.

Exposure unit is joule per cubic meter but the most practical unit for occupational exposure measurements is the WL Month WLM. 1 WLM equals 1WL × 173 h because the working time per month is set of 173 h.

## Dose

### Dose received due to radon

Exposure to radon in certain tissues or whole body can be estimated from experimental measurements after calculating total PAEC. On the other hand, effective dose is calculated by properly employing Dose Conversion Factors (DCFs) (30–34). DCF determination is a very complicated process running with two different approximations as is explained hereafter.

#### Epidemiological approach

Following the results from several epidemiological investigations, various DCFs have been proposed so-far. United Nations Scientific Committee on the Effects of Atomic Radiation (35) proposed the DCF of 0.17 nSv/Bq h m^−3^ regarding the exposure to ^222^Rn and the DCF of 9 nSv/Bq h m^−3^ regarding the exposure to EEDC_222_. ICRP 65 proposed the DCF of 4 mSv/WLM for exposure indoors and the DCF of 5 mSv/WLM for occupational exposure. Both DCFs do not account for the energy fraction carried by free progeny (1).

#### Dosimetric approach

According to this approach, various DCFs are estimated *in vitro* from human respiratory track models. The way of precise calculations of several environmental and personal factors could be taken under account. Factors of great importance are the aerosol distribution into ambient air (36), the inspiration rate, and the size of lungs. A human respiratory track model was presented by ICRP report No 66.

Dose delivered to bronchial tissues, DB, is defined as:

$$D_{B}=E\left[f_{p}D_{u}+\left(1-f_{p}\right)D_{a}\right]\left(3.\mathrm{32}\right)$$

where *D_u_* and *D*_α_ are the values of dose received per unit of exposure both from unattached and attached fraction, respectively (37). *D_B_* is affected significantly by the variations of *fp*. Since the aerosol size distribution significantly affects *fp* (29), *D*_α_ depends on aerosol sizes and consequently on ambient air conditions. *D_u_* is usually one order of magnitude lower than Da.

### Epidemiology

In 1988, the International Agency for Research on Cancer (IARC) classified radon as an A-level human lung carcinogen, based on the epidemiological studies of underground miners. In parallel, several large-scale residential epidemiological studies were launched in an effort to investigate factors possibly affecting dose–response relationship due to residential radon. Some included pooled analysis. Different case–control studies: (a) conducted face to- face interviews both for patients with lung cancer and hospital controls or (b) employed questionnaires to analyze the risk of lung cancer in relation to domestic radon exposure adjusting for tobacco consumption. Since 2000, several joint analysis studies have been published, integrating the basic individual data from cases and controls, and applying standard methodologies in defining selection criteria and statistical analysis. These studies showed that there is a linear correlation between risk for lung cancer and cumulative radon exposure. The latency period identified is between 30 and 35 years. The slope of the linear exposure–response relationship ranges between 1.08 and 1.13 per 100 Bq/m^3^.

Recent studies proposed various scenarios of dose-affecting environmental factors. Two very significant studies were carried out by Porstendörfer (28, 29). These indicated linear correlation between DCFs and *fp* factor. A new factor introduced with the term “environmental factor,” which stands for the aerosol particle distribution in ambient air that strongly (directly) affects *fp* value. Another term introduced was the “individual factor,” which accounts for the size of the lungs (i.e., age), the rate and mode of breathing (nasal or oral, or quota between these two forms). It is emphasized that previous models utilized higher values for DCFs because workers breathe rather orally than from nose. In Porstendörfer (28, 29), the dose per exposure unit of short-lived radon progeny, was calculated using dosimetric approach. Calculations were based on a lung dose model with a structure that is related to the respiratory tract model proposed by ICRP in the report No66. The dose relevant parameters, unattached fraction (*fp*), and size distribution of the unattached and aerosol attached radon decay products for different living and working places were reported. Taking into account these characteristics, the effective dose per exposure unit and the conversion factor (DCF), of the radon decay products was estimated.

**Figure 2:**
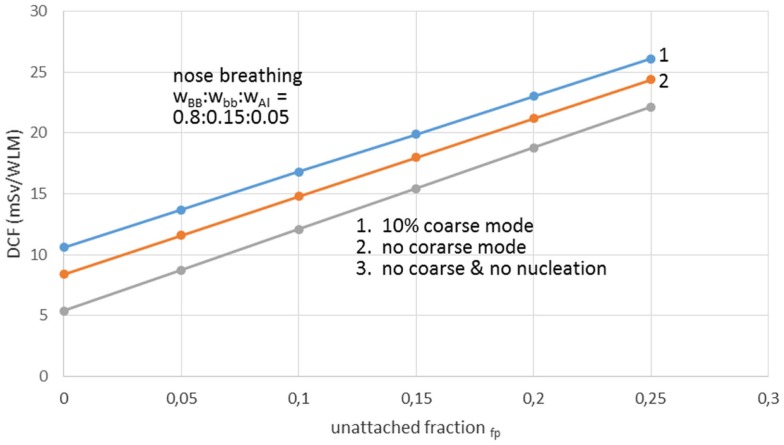
**The dose conversion factor (DCF) as function of the unattached radon progeny clusters for working places with different aerosol conditions**. Reproduced from data reported by Porstendörfer (29).

**Figure 3:**
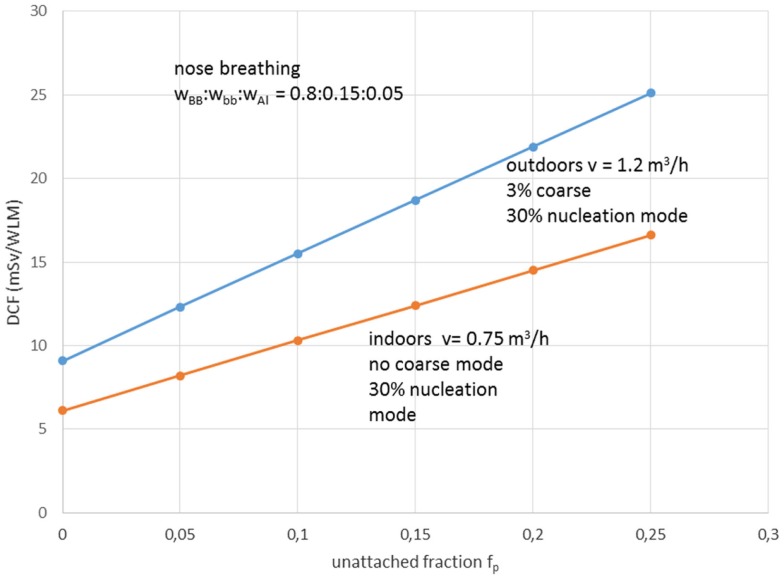
**The dose conversion factor (DCF) as function of the unattached radon progeny clusters in indoor and outdoor air**. wBB, wbb, and wAl are the relative cancer sensitivity distribution of the bronchial, bronchiolar, and alveolar regions of the thoracic lung, respectively, and *v* = inhalation rate. Reproduced from data reported by Porstendörfer (29).

Aerosol size distribution can be described as the sum of three-modal phases, or more strictly, the sum of three independent size log-normal distributions (38). The three-modal aerosol phases from Porstendörfer (29) are: (1) nucleation mode or *n*-mode with arithmetic mean diameter (AMD) 30–40 nm; (2) accumulation mode or a-mode with AMD 250–450 nm; and (3) coarse mode or c-mode with AMD 2000–6000 nm. Porstendörfer (28) reported DCF values ranged between 6 and 39 mSv/WLM. Porstendörfer (29) collected and reported data for distributions of aerosol particles from measurements in many residences and working places (Figure 2) as well as from outdoor air. For indoor and outdoor air, Porstendörfer illustrated correlations between DCF and *fp* factor (Figure 3).

### Exposure and dose of workers

#### For miners

The results from combined analysis epidemiological studies among various cohorts of miners showed that the excess relative risk ranges from 0.49 to 1.6 per 100 WLM. Some characteristics of the cohorts may explain variations in the relative risk, including duration of follow-up, attained age, duration of work, exposure levels, and background rates of lung cancer (39).

#### For water workers

**Figure 4:**
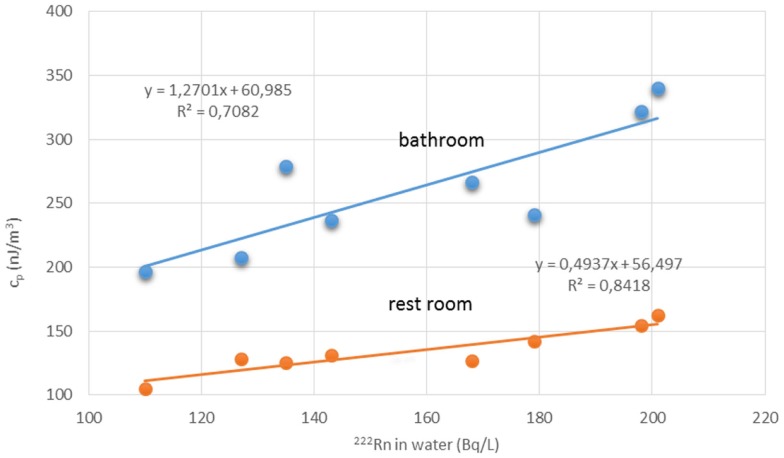
**Radon concentration in water samples versus PAEC measured in two different spa premises**. Reproduced from data reported in Vogiannis et al. (54).

**Figure 5:**
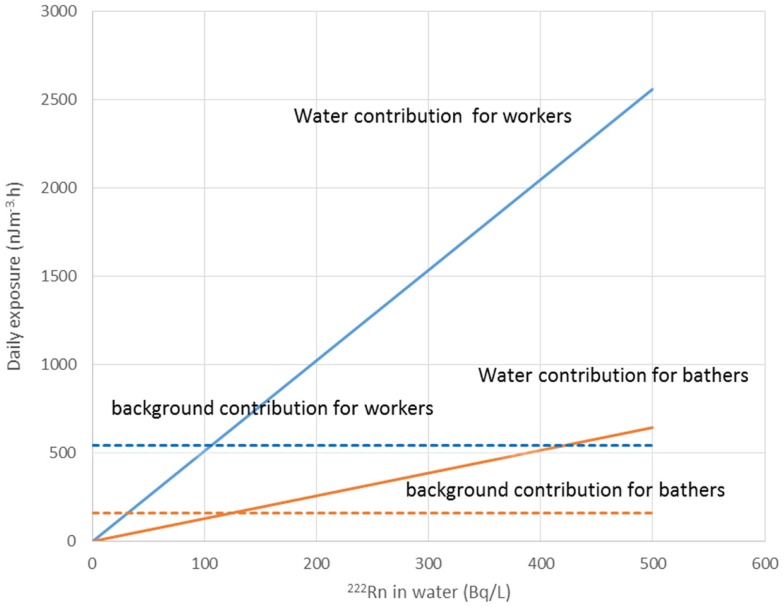
**Daily exposure in PAEC correlated with radon in water**. Exposure was estimate for works and bathers taking into account the time spent in the various spa premises. Reproduced by data reported in Vogiannis et al. (54).

**Figure 6:**
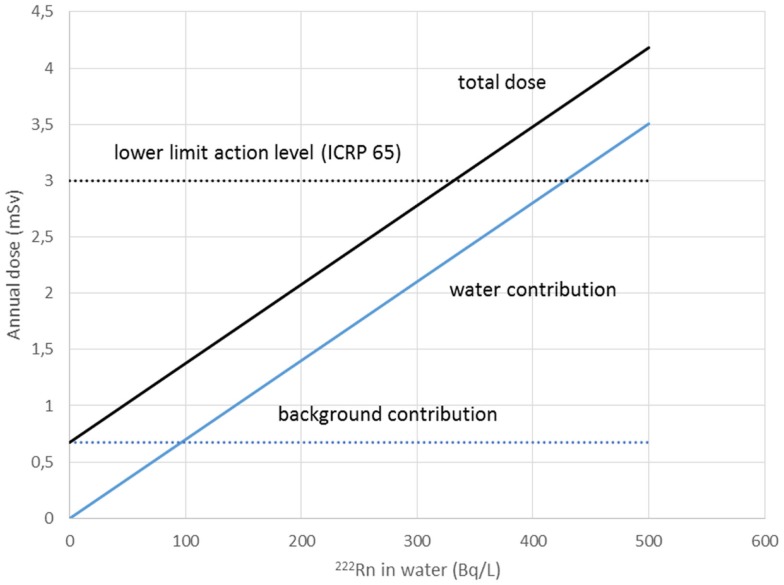
**Annual dose received by spa workers correlated with radon in water**. Slope corresponds to the pattern of use of the investigated spa. Safety norms could be easily derived from such linear regressions. Reproduced by data reported in Vogiannis et al. (54).

Water workers, especially spa workers, are not to be neglected because of the renewed interest in the use of spas. Many historic facilities are reconstructed or revived on purpose of alternative therapy and tourism. Among these economic activities, the thermal baths feature prominently in the interest of investors. Results from various researches for radon content in water, radon environment in spa, and other facilities, were reported estimations of exposure and doses received from workers and public (Table 1). Early measurements in the Herculane Spa region showed high radon concentrations (up to 7.4 MBq/m^3^) for the emanated gases, which accompany geothermal waters originating in the hydrogeothermal deposit existing in this area (40). Natural radioisotopes, which exist in the waters of the municipal thermometalic radioactive springs (spas) of the Greek island Ikaria, were measured directly in collected water samples, using a 3 × 3 inch NaJ detector. Reported ^222^Rn concentrations ranged from 0.18 to 30.48103 Bq/m^3^ (41). Recent research in Ikaria spas (27) indicated average concentrations of radon in water from (10.7 ± 1.4)*0.102 Bq/L for the Spilaion spas to (22.0 ± 1.4)*0.102 Bq/L for the Apollon spas. Calculated effective doses ranged between 0.001 and 0.589 mSv/year for patients and between 0.001 and 18.9 mSv/year for workers. Apollon spas presented quite higher doses. These values were the highest reported for Greece, and on the other hand are significant worldwide. Two are the major reasons for the significant amounts of ^222^Rn released inside the spa air: (1) the manner in which water enters spa and (2) its high temperature, both providing explanations for high exposures of workers and visitors (42). Furthermore, in samples collected from 23 springs and 4 taps in health resorts of the Klodzka valley in the Studety Mountains in Poland, A.M activity values of ^222^Rn were found between 0.18 Bq/L and 1332.8 ± 28.0 Bq/L (43). In the Badgastein spa, Austria, a survey conducted over a 15-month period reported a number of continuous radon activity measurements in various spa facilities, and for different types of therapy. For assessing the occupational exposure, a combination of long- and intermediate-term integrating radon measurements and continuous radon and progeny measurements was used to investigate different treatment facilities. Dose calculations based on the PAEC measured values showed estimations for the effective annual doses between 14 and 48 mSv, exceeding the action level recommended for workplaces in ICRP 65 (3–10 mSv) (44). Different series of samples were collected and measurements were made for the radon concentrations in water from 54 spas and in air into 20 spas. In six cases of the latter group, the air radon concentration was studied in different working areas occupied by the employes. The radon concentrations in water were found between 2 and 775 × 10^3^ Bq m^−3^. The radon concentrations in air were found between 10 and 5,200 Bq m^−3^. The latter were used to estimate the dose received by each occupational group in the spa by taking into account the radon concentration in their main working area. Using a DCF factor of 1.43 Sv/J h m^−3^, the estimated effective doses were found to range between 1 and 44 mSv year^−1^. This upper limit is higher than the recommended annual limit of 20 mSv year^−1^ for the occupational dose (45). 1 L ground water samples were selected from more than 500 water supply facilities. Samples exposed to track-etch detectors in order to get the mean radon concentration of the main working places. In addition, for a period of 2 months, the personnel had to wear a track-etch detector while they were in the supply facilities in order to get an estimate of their individual exposure level. In the east Bavarian crystalline region, indoor radon gas concentrations of up to 300 kBq/m^3^ were observed. About 10% of the processing plant workers of this region get an annual effective dose of more than 20 mSv (46). Hot spring and mineral water from an extended region in Slovenia surveyed ^222^Rn concentrations in water samples. The reported values ranged from 5 to 62.9 Bq/L (47). Another survey in Spanish spas was carried out in 54 spas and in their premises. Concentration of ^222^Rn dissolved in collected water samples was found as high as 824 Bq/L. 25 samples were found with radon concentration below 25 Bq/L, 15 with concentrations between 20 and 150 Bq/L, 7 with concentrations between 150 and 300 Bq/L, and 7 with higher values. Radon concentration in the air was measured inside the spa at different sites, close to the spring, inside the bath, shower, inhalation room, and entrance hall. Measured concentrations ranged from 3560 to 6650 Bq/m^3^ from the different sites. Estimated doses delivered were 200 mSv/year to the bronchial epithelium and 24 mSv/year to the total body. Doses estimated for Spanish spas were considered for a working period of 10 weeks per year because spa is open only for the summer months (48). Another survey was carried out in the health resorts of the Sudety Mountains in Poland. Groundwater collected from different depths into a borehole reported ^222^Rn content with concentrations up to 3000 Bq/L. The most significant finding from this work was that ^222^Rn concentration in water decreases rapidly as the depth increases to about 100 m; below this depth, it is almost constant to the depth of 600 m (49). Moreover, values for ^222^Rn concentrations in water reported from groundwater used for household supply purposes were high enough, considering the water stations prior the water distribution network. In Brazil groundwaters, the dissolved radon ranged between 0.1 and 122 Bq/L, being log-normally distributed, with a modal value of 49 Bq/L. Experiments for ^222^Rn leakage from sandstones yielded a theoretical value of 51 Bq/L for ^222^Rn in water, showing that theoretical modeling can reliably be used to interpret laboratory and field data (50). Those values could be motivated high radon concentrations in indoor air, if the large amount of pumped water was collected in tightly closed water stations. For the same reason, probably, a high risk was identified in a case of China, where in 282 measured samples, ^222^Rn concentrations in groundwater ranged between 0.71 and 3735 kBq/m^3^, with a geometric mean of 147.8 kBq/m^3^ (51). ^222^Rn concentrations in medicinal groundwater of Ladek Zdrój (Poland) showed mean values between 134 and 1284 Bq/L. (52). Effective dose values for cave and spa personnel generally did not exceed the 20 mSv/year, the limit recommended for workers by the ICRP 60 publication. In cases where calculated dose values were higher, it was recommended to implement more effective ventilation systems or to reduce the occupancy into high radon atmospheres. Radiation doses received from visitors and patients were one or two orders of magnitude lower than those received from the personnel (53). Previous studies did not attempt to investigate the correlation between the radon content of the water versus the exposure or dose delivered. A semi-experimental method was proposed by Vogiannis et al. (54). This method was composed from a series of ^222^Rn and progeny measurements to estimate PAEC on the basis of collected radon concentration measurements in water samples (Figure 4). A linear correlation was found between PAEC released into the spa air and Radon concentration in water (Figure 5), same curves were produced for the correlation of dose delivered by users and radon concentration in water (Figure 6). The slope of the linear regression in Figure 4 is unique for every investigated premise, and strongly depends on the pattern of water-usage followed. Therefore, exposure and dose could be actually estimated only from measurements of radon in water. The water contribution to the daily exposure for workers and bathers was calculated by adopting a typical scenario of bath use. The daily exposure of a worker was assumed to be a combination of background radiation (8 h), time spent in bathroom (2 h), and time spent in restroom (6 h). A bather was assumed to spend 20 min in bathroom and about 2 h in restroom for shower, therapy, and dressing. Figure 5 shows the water contribution to the daily exposure for a worker and for a bather according to the above scenario of use. The horizontal lines show the exposure due to background ^222^Rn concentration. The daily exposure in spa facilities is the summary of the two lines referred to each occupant of the bath. Figure 6 shows the correlation between annual doses received by spa workers with radon content in bath water. The slope of each curve is unique and corresponds to the pattern of use of the investigated spa. Eventually, this approach (dynamic) could be adapted and applied to other facilities where water is the dominant source of ^222^Rn release in air. Safety norms could be easily derived from such linear regressions.

**Table 1:** **Data from water places**.

Region		Rn in water (Bq/L)			Rn in air (Bq/m^3^)	Dose	Reference
		Min	Max	Mean	
Herculane spa					<7.4 × 10^6^		(39)
Ikaria Island Greece					0.18–30.48.10^3^		(40)
	Apollon spa			(22.0 ± 1.4) 0.102		0.001–0.589 (mSv/year) for patients	(27)
	Spilaion spa			(10.7 ± 1.4) 0.102		0.001–18.9 (mSv/year) for workers	
Sudety Mountains, Poland	Klodzka valley	0.18	1332.8 ± 28.0				(43)
Badgastein, Austria		2	775		10–5,200	14–48 mSv/year occupational, based on the PAEC measured values	(44)
						1–44 mSv/year conversion factor of 1.43 Sv/J h m^−3^	
Bavarian crystalline region						>20 mSv 10% of the processing plant workers	M. (46)
Extended region in Slovenia				5–62.9			(47)
Spanish spas		20	824		3560–6650	200 mSv/year to the bronchial epithelium and 24 mSv/year to the total body	(48)
Sudety Mountains in Poland	Radon content in groundwater		3000				(49)
Groundwaters in Brazil		0.1	122	Log-normally distributed, with a modal value of 49 Bq/L			(50)
China groundwater		0.71	3735	Geometric mean 147.8 kBq/m^3^			(51)
Medicinal groundwater of Ladek Zdrój (Poland)		134	1284				(52)

*Radon concentration in water samples from groundwater and spa waters collected from various regions across the world. In some cases, there are values of radon concentration in the indoor air of spa premises Column 6. Measurements of dose delivered by spa workers or other water workers are also presented*.

## Risk Assessment

As already mentioned, lung cancer rates in miners have been studied using a cohort design. In these studies, all miners were identified during a particular time period. Then the persons were followed over time, regardless of whether these still remain employed. The vital status of each person was established at the end of the follow-up period. For dead persons, the date and cause of death was ascertained. Then the death rate from lung cancer was calculated, both overalls and after adding factors such as age, calendar period, and cumulative exposure to radon (21, 55). It is noted that in cohort studies, the exposure to radon was usually estimated retrospectively. Moreover, in many studies the quality of the exposure assessment was low, particularly in the early years of mining, when the exposures were highest and no radon measurements are available (21). In addition, radon progeny concentrations were also not available (21). According to the report of World Health Organization (21), the risk of lung cancer observed in underground miners due to exposure on radon, was the main reason to investigate similar risk for the general population, due to radon exposure in houses, working places, and buildings. However, there are significantly different conditions in mines and indoors. As a result, great uncertainty was observed when data from miner studies extrapolated for the assessment of the risk of lung cancer from radon in houses/buildings. Initial attempts to study the risk of lung cancer from indoor radon (known as ecological studies) examined the correlation between average radon concentration and average lung cancer rates in different geographical areas. Ecological studies did not provide us with adequate information of the radon related risk. Later, case–control studies which investigate the correlation between lung cancer risks and residential radon for a predetermined number of individuals who have developed lung cancer, together with a predetermined number of control individuals who have not developed the disease. For the most of these studies have not been a sufficient target group to provide clear evidence that an increased risk existed. In order to compare those findings it is necessary to assemble the data on radon concentration, smoking history, and other relevant factors for each individual in each of the original studies and to collate them in a uniform way. Three analyses collating and comparing the individual information from a number of component studies have now been carried out, including 13 European studies, 7 North American studies, and 2 Chinese studies, respectively. There is clear evidence that radon is a lung carcinogenic compound in the general population at concentrations found in ordinary homes. It was found that there was no evidence for the proportionality between increases in risk per unit increase in radon concentration varied with the age, sex, or smoking habits of the study subjects more than would be expected by chance. There is a linear relationship for the dose–response curve, with no evidence of a threshold, and there was substantial evidence of a risk increase even below 200 Bq/m^−3^, the concentration at which action is required in many countries.

## International Awareness (Norms and Legislation)

European Atomic Energy Commission (EURATOM) (56) recommended to its member countries to establish an appropriate system for reducing any exposure to indoor radon concentrations. Within this system adequate public information and response to public concern deserve particular attention. Below significant information is repeated.

COMMISSION RECOMMENDATION of 21 February 1990 on (57):

1. An appropriate system be established for reducing any exposure to indoor radon concentrations. Within this system adequate public information and response to public concern deserve particular attention.
2. For existing buildings:
  (a) a reference level for consideration of remedial action be used: where exceeded, it should be cause for consideration of simple but effective measures aimed at reducing the radon level;
  (b) the reference level be an effective dose equivalent of 20 mSv per annum, which for practical purposes, may be taken as equivalent to an annual average radon gas concentration of 400 Bq/m^3^;
  (c) the urgency of the remedial action take account of the extent to which this reference level is exceeded;
  (d) where remedial actions are considered necessary, the public concerned be informed on the radon levels it is exposed to and on the remedies available to reduce such levels.
3. For future constructions:
  (a) a design level be used to aid the relevant authorities in establishing regulations, standards, or codes of construction practices for circumstances under which the design level might otherwise be exceeded; the design level be an effective dose equivalent of 10 mSv per annum, which for practical purposes, may be taken as equivalent to an annual average radon gas concentration of 200 Bq/m^3^;
  (b) information be provided to those involved in the construction of new buildings, as relevant, on possible radon exposure levels, and on preventive measures which could be taken.
4. When remedial or preventative measures are being determined, the principles of optimization be applied in accordance with the Community basic safety standards (4).
5. Because of diurnal and seasonal variations of indoor radon levels, radiation protection decisions should in general be based on the annually averaged measurements of radon gas or daughters in affected buildings using integrating techniques. The competent authorities should ensure that the quality and reliability of measurements are adequate.
6. Criteria be developed for identifying regions, sites and building characteristics likely to be associated with high indoor radon levels. Investigation levels for the underlying parameters (i.e., activity in soil and building materials, permeability of ground, etc.) could be used to identify such exposure circumstances.

In this recommendation reference levels are proposed in Becquerel per cubic meter for the purpose of the convenience of existing measurement techniques. The recommended levels for existing and new buildings are quite high, especially for existing constructions, probably the reason for that was the high cost of the improvements needed to be done.

Much later in 1996 European Council returned in Radon and Thoron (now) doses included them in a general directive for dangers from ionizing radiation (58).

From ANNEX III C:

“For radon progeny and thoron progeny the following conventional conversion factors apply, effective dose per unit potential alpha-energy exposure (Sievert per Joule hour per cubic meter):

Radon at home: 1,1

Radon at work: 1,4

Thoron at work: 0,5

Potential alpha energy (of radon progeny and thoron progeny): The total alpha energy ultimately emitted during the decay of radon progeny and thoron progeny through the decay chain, up to but not including ^210^Pb for progeny of ^222^Rn and up to stable ^208^Pb for progeny of ^220^Rn. The unit is J (Joule). For the exposure to a given concentration for a given time, the unit is Joules hour per cubic meter.”

However, it is clear that the overall intake dose limit for the general public adjusted at 1 mSv per year

“… The limit for effective dose shall be 1 mSv in a year. However, in special circumstances, a higher effective dose may be authorized in a single year, provided that the average over five consecutive years does not exceed 1 mSv per year. ”

and for workers at an average of 20 mSv per year.

“… The limit on effective dose for exposed workers shall be 100 millisieverts (‘mSv’) in a consecutive five-year period, subject to a maximum effective dose of 50 mSv in any single year. Member States may decide an annual amount.” But, in any case “… Each Member State shall take reasonable steps to ensure that the contribution to the exposure of the population as a whole from practices is kept as low as reasonably achievable (ALARA), economic and social factors being taken into account. The total of all such contributions shall be regularly assessed.”

## Conclusion

Undoubtedly, radon is a health hazard but not by itself. Manmade constructions or other activities should be a potential factor or increased concentrations of radon and progeny, especially in spaces without adequate air exchange. Many reports illustrate the elevated radon concentrations in working places, spas, caves, and houses, due to small aeration or constructive details or concentration of radon bearing underground water. All concentrations could be lowered by small improvements or interventions made by specialists. Sufficient aeration appears to be the crucial factor of radon levels reduction in houses. Nevertheless, there are many mechanical ways to keep the indoor radon levels low, whether there is contradiction to the energy loses. Great importance should be addressed to working places, such as mines and other premises – especially spas – due to the high radon levels recorded from many research works all over the world. Such places must be subjected to special investigation, taking into account the whole mobility of radon and progeny in the ambient air. However, the general public ignorance for radon issues appears to be the major problem. Governments have a duty to proceed with mapping of areas that may be designated as high risk. This will facilitate the process of legislating constructive rules for occupational activities in high risk areas.

## Conflict of Interest Statement

The authors declare that the research was conducted in the absence of any commercial or financial relationships that could be construed as a potential conflict of interest.
